# Dietary Antioxidants and Metabolic Diseases

**DOI:** 10.3390/ijms222212558

**Published:** 2021-11-22

**Authors:** Alessandra Durazzo, Ginevra Lombardi-Boccia, Antonello Santini, Massimo Lucarini

**Affiliations:** 1 CREA—Research Centre for Food and Nutrition, Via Ardeatina 546, 00178 Rome, Italy; 2 Department of Pharmacy, University of Napoli Federico II, Via D. Montesano 49, 80131 Napoli, Italy

**Introduction**

Considering the change in people’s diets and lifestyle, the number of people with metabolic diseases such as diabetes, obesity and gout is on the rise. Some studies have shown that dietary antioxidant nutrients (e.g., ascorbic acid, β-carotene, vitamin E and selenium) can have preventive and therapeutic effects on some metabolic diseases. Moreover, antioxidants are substances that may protect cells against free radicals, which may play a role in health conditions [1,2,3].

Nonetheless, the real impact of these dietary antioxidants and their mechanisms of action is far yet to be completely elucidated. Preclinical and/or human evidence are essential elements. Many foods in the human diet, such as vegetables and fruits, contain antioxidants that may act interactively, even synergistically, with the endogenous antioxidant defense system to restore or maintain redox homeostasis. Some phytochemicals can influence the pathways of molecular signal transduction such as inflammation cascades, metabolic disorders and oxidative stress.

The treatment of metabolic diseases includes a healthy and balanced diet, and supplementation with nutraceuticals [4,5,6,7,8,9,10,11,12].

To give a current snapshot of the interest raised in the international research context within this topic, a search throughout the Scopus online database was carried out by means of a string TITLE-ABS-KEY (“dietary antioxidant*” AND “health*”). The “full records and cited references” were exported and processed using the VOSviewer software (version 1.6.16, 2020; www.vosviewer.com, accessed on 6 November 2021) [13,14,15]. The search returned 822 publications covering the time range from 1973 to 2022, and a total of 830 terms were identified and visualized as a term map in Figure 1.

Figure 1 allows us to identify the main terms correlated with research on the relationship between dietary antioxidants and health and identifies the main existing research lines focused on this topic. It is interesting to observe that, among the top-recurring keywords, antioxidants, humans, male, female, adult, ascorbic acid, alpha tocopherol, oxidative stress, diet, dietary intake and antioxidant activity appear. The most cited paper (2271 times) is the review by Pandey and Rizvi [16] on plant polyphenols as dietary antioxidants in human health and disease.

This Special Issue is focused on recent advances in the study of the health benefits of dietary antioxidants in metabolic diseases as well as on the discovery of novel molecular therapeutic mechanisms and the testing of novel targeted therapies.

A current challenge is given by combing through databases, repositories, and infrastructures to link information and compositive data on antioxidant to metabolomic pathways and biomarkers from the perspective of interoperability [17,18,19].

## Figures and Tables

**Figure 1:**
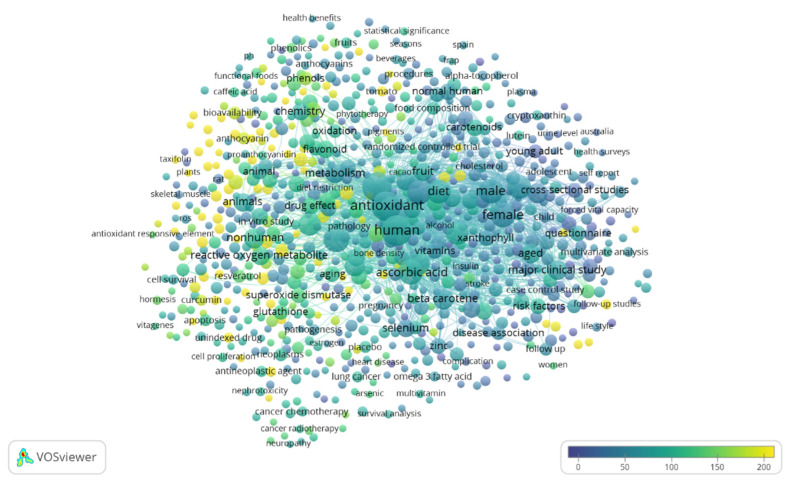
Term map for the relationship between dietary antioxidant and health research. Bubble size represents the number of publications. Bubble color represents the citations per publication (CPP). Two bubbles are closer to each other if the terms co-appeared more frequently (bibliometric data were extracted from the Scopus online database and elaborated by the VOSviewer software).
